# Automated Diet Capture Using Voice Alerts and Speech Recognition on Smartphones: Pilot Usability and Acceptability Study

**DOI:** 10.2196/46659

**Published:** 2023-05-16

**Authors:** Lucy Chikwetu, Shaundra Daily, Bobak J Mortazavi, Jessilyn Dunn

**Affiliations:** 1 Department of Electrical and Computer Engineering Duke University Durham, NC United States; 2 Department of Computer Science and Engineering Texas A & M University College Station, TX United States; 3 Department of Biomedical Engineering Duke University Durham, NC United States

**Keywords:** automatic dietary monitoring, ADM, food logging, diet logging, voice technologies, voice alert, speech recognition, natural language processing, NLP

## Abstract

**Background:**

Effective monitoring of dietary habits is critical for promoting healthy lifestyles and preventing or delaying the onset and progression of diet-related diseases, such as type 2 diabetes. Recent advances in speech recognition technologies and natural language processing present new possibilities for automated diet capture; however, further exploration is necessary to assess the usability and acceptability of such technologies for diet logging.

**Objective:**

This study explores the usability and acceptability of speech recognition technologies and natural language processing for automated diet logging.

**Methods:**

We designed and developed base2Diet—an iOS smartphone application that prompts users to log their food intake using voice or text. To compare the effectiveness of the 2 diet logging modes, we conducted a 28-day pilot study with 2 arms and 2 phases. A total of 18 participants were included in the study, with 9 participants in each arm (text: n=9, voice: n=9). During phase I of the study, all 18 participants received reminders for breakfast, lunch, and dinner at preselected times. At the beginning of phase II, all participants were given the option to choose 3 times during the day to receive 3 times daily reminders to log their food intake for the remainder of the phase, with the ability to modify the selected times at any point before the end of the study.

**Results:**

The total number of distinct diet logging events per participant was 1.7 times higher in the voice arm than in the text arm (*P*=.03, unpaired *t* test). Similarly, the total number of active days per participant was 1.5 times higher in the voice arm than in the text arm (*P*=.04, unpaired *t* test). Furthermore, the text arm had a higher attrition rate than the voice arm, with only 1 participant dropping out of the study in the voice arm, while 5 participants dropped out in the text arm.

**Conclusions:**

The results of this pilot study demonstrate the potential of voice technologies in automated diet capturing using smartphones. Our findings suggest that voice-based diet logging is more effective and better received by users compared to traditional text-based methods, underscoring the need for further research in this area. These insights carry significant implications for the development of more effective and accessible tools for monitoring dietary habits and promoting healthy lifestyle choices.

## Introduction

Diet-related diseases such as type 2 diabetes and coronary heart disease continue to increase at a staggering rate [1,2]. The International Diabetes Federation estimates that every 5 seconds, someone dies of diabetes or diabetes-related complications [3], and according to the World Health Organization, obesity prevalence around the world has nearly tripled since 1975 [4,5]. These global data highlight an urgency for innovative solutions to alleviate this problem. Incentivizing the adoption of a healthy diet can be instrumental in achieving positive outcomes [6-8]. Consequently, there have been many conversations concerning potential policy approaches such as taxing sugar-sweetened beverages [7,8] or reducing sodium levels [6,8] in processed foods to influence individuals’ adoption of a healthy diet. As for individualistic approaches, a proliferation of studies [9] demonstrates that diet monitoring carries substantial promise because it brings mindfulness [10] to eating. Mindfulness allows individuals to not only be aware of what they are eating but also identify when they are eating, food intolerances, positive and negative dietary habits, and potentially accelerated efforts toward a healthy lifestyle.

Gold-standard diet monitoring approaches include manual self-reporting methods such as 24-hour dietary recall, which are often highly inaccurate [9] and not user-friendly [9], leading to user attrition. In light of the shortcomings of conventional methods, there has been a growing interest in automatic dietary monitoring [9,11,12]. Automatic dietary monitoring systems leverage technology to monitor relevant aspects of food intake, such as timing and duration of meals, amount of food consumed, and nutritional content. In recent years, advances in mobile technologies have facilitated the proliferation of smartphone-assisted food-logging applications, such as text-based applications that rely on user-inputted text and computer vision-based applications that rely on barcode scans or photos taken by users to support food recognition and calorie estimation [13-15]. However, smartphone-assisted diet monitoring technologies suffer from low adherence [16] mainly because it is challenging for many individuals to remember to log their food intake [17]. Even with automated reminders and notifications, adherence remains low because the average user receives many other notifications from applications on their device [18], leading to alert fatigue. Furthermore, most of the alerts that individuals receive on their smartphones are silent or have generic reminder tones, which rarely attract the user’s attention. Diet logging is also often perceived as time-consuming [16,17], primarily because of the need for hands-on manual text or image and photo input, and the time required to retrieve each food item’s nutritional content. Accordingly, even with smartphone assistance, there is still a low long-term uptake of food-logging applications.

Accordingly, diet logging continues to be a challenge despite the existence of powerful technologies such as voice alerts, speech recognition (use of computers to detect human speech with the ultimate goal of translating it into text), and natural language processing (NLP—providing computers with an ability to parse and analyze human speech or text) that could change the landscape but have been less explored in this area [19-21]. This is especially important for improving diet recall and diet logging frequency, which could lead to more effective research and nutritional interventions for diet-related diseases such as type 2 diabetes.

This paper explores the usability and acceptability of speech recognition technologies for automated diet logging. We define *automated diet logging* as the act of acquiring a user’s spoken or typed natural language description of their consumed food, and using this to generate a time-stamped diet log comprising food item representations and automatically retrieved nutritional content (supported by NLP). We have developed base2Diet, an iOS application that employs voice-assisted technologies for hands-free diet logging. Some critical questions we strive to answer with base2Diet include the following: does voice-assisted diet logging improve diet logging adherence? Are users open to voice-assisted diet logging?

## Methods

### Application Development

**Figure 1:**
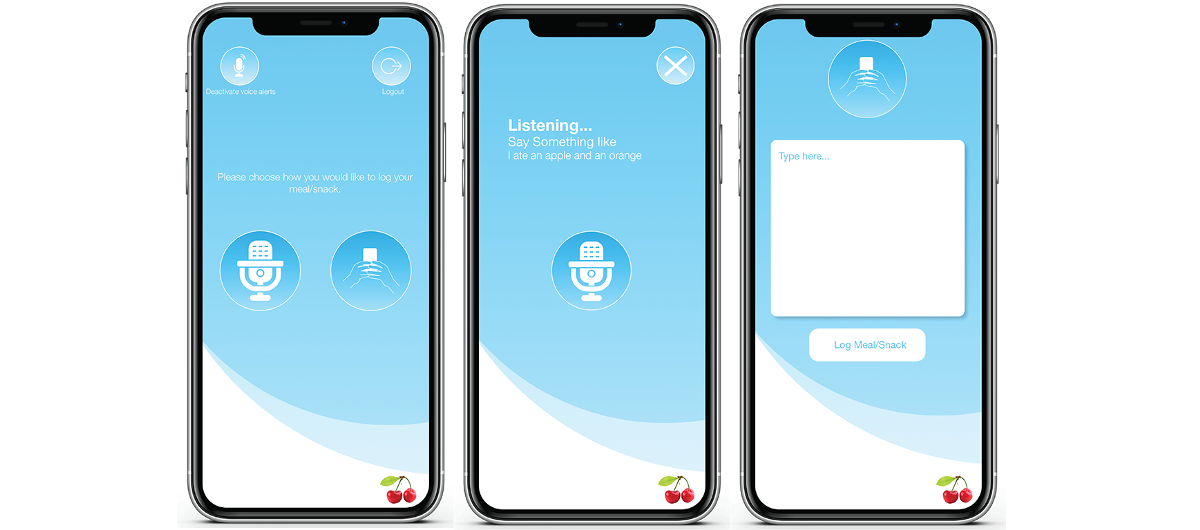
Screenshots of the base2Diet app. The leftmost screen shows the voice version of the app. It allows individuals to choose how they would like to log their food intake. The microphone button indicates choosing voice to log food intake, and the texting hand indicates choosing to text for diet logging. If an individual chooses voice, the middle view is presented. If the user chooses text, the rightmost view is presented.

**Figure 2:**
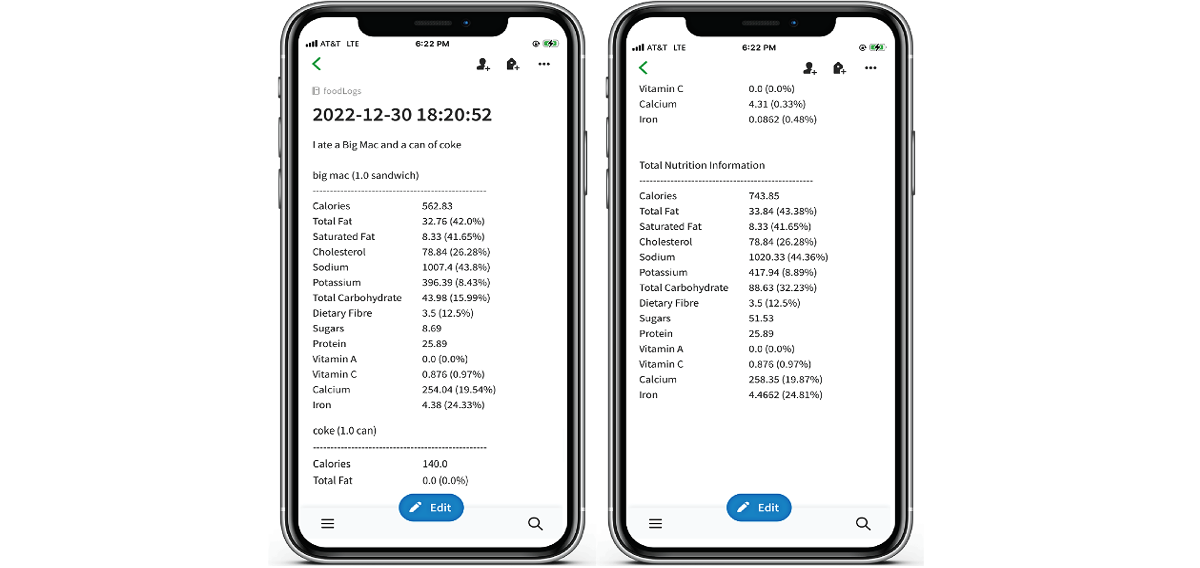
Sample timestamped diet log automatically created by the base2Diet application in Evernote.

**Figure 3:**
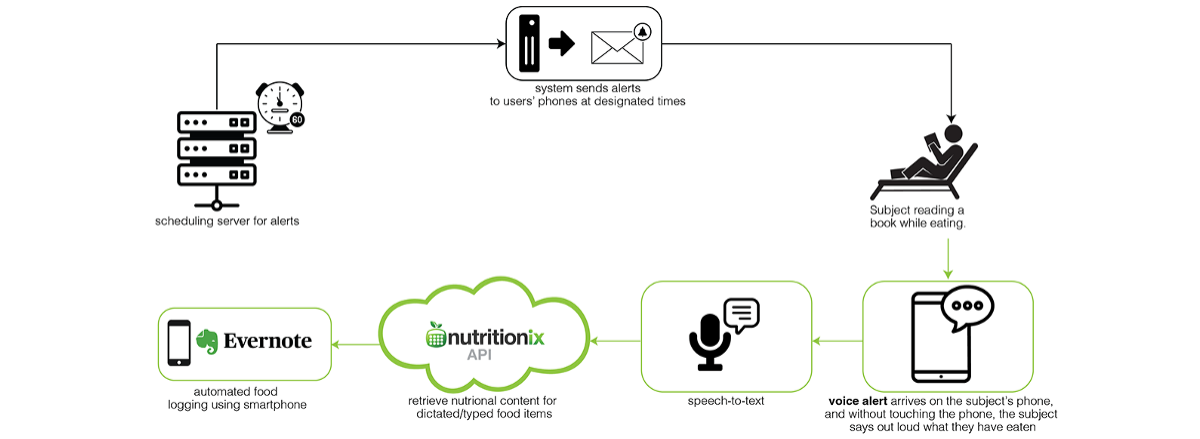
base2Diet app system diagram.

We designed and developed base2Diet—an iOS smartphone application (Figures 1-3) for voice-assisted diet logging using Swift programming language on XCode—Apple’s integrated development environment. The app has 2 modes: text and voice. Text mode uses personalized text alerts to prompt users to log their food intake using text. In contrast, voice mode uses simultaneous text notifications and voice alerts to prompt users to log their food intake, which they can do using either text or voice. To send personalized alerts to users’ phones at designated times, we designed and developed a Node JS scheduling server hosted on Google Cloud. In addition, we used Firebase for user authentication and cloud storage. The app automatically creates time-stamped diet logs (Figure 2) based on user-provided natural language descriptions (voice or text) of the consumed food and the food’s nutritional content, which it obtains by querying Nutritionix [22]—an NLP-based application programming interface (API) that has been used in health applications such as the Vida Health app [23], and in several research studies [24,25]. Nutritionix’s NLP API allows users to describe their meals in everyday language and receive an accurate breakdown of the nutritional information of the consumed foods. For example, by simply stating, “I had 5 bacon slices, 1 plain bagel, a fried egg, a slice of cheddar cheese, and a glass of milk for breakfast,” the Nutritionix API will search for 5 items: *bacon* (5 slices), *plain bagel* (1), *fried egg* (1), *cheddar cheese* (1 slice), and *glass of milk* (1) and provide nutritional content for each of them based on the provided quantities. Conventional databases lacking NLP functionality would not understand that this query requires the retrieval of 5 distinct items from the database. To facilitate voice-assisted diet logging, we used Apple’s speech recognition framework [26] to detect and transcribe speech to text. Finally, we used the Evernote API [27] to connect base2Diet with Evernote, which we used for creating and storing time-stamped diet logs.

### Study Design

We performed a 2-arm study with 18 participants (text: n=9, voice: n=9) and no crossover. We used a combination of self-selection and random sampling to allocate participants into groups. Strong preferences expressed by participants for a particular study arm were honored; otherwise, participants were randomly assigned to one of the study arms. Participants in the text arm only used text for diet logging, and they received personalized text alerts as reminders to log their food intake. Participants in the voice arm could use both voice and text to log their food intake, and they received personalized, simultaneous text notifications and voice alerts as reminders for them to log their food intake. Voice-assisted diet logging in the base2Diet app is both interactive and personalized. Once a user in the voice arm chooses to log their food intake using voice, the base2Diet app waits for the user to speak. If user’s speech is not detected within 8 seconds, the app says out loud, “Sorry X, I didn’t quite get that. Please try again,” where X is the user’s name. After this message, if no user’s speech is detected for another 4 seconds, the app closes the session and emits a failure notification sound. If speech is detected, base2Diet transcribes the speech to text, uses the transcribed speech text to query Nutritionix for the stated foods’ nutritional content, and automatically creates a time-stamped Evernote diet log comprising the transcribed speech text and the retrieved nutritional content (Figure 2). The base2Diet app displays the natural text transcribed from the user’s speech (Figure 1). If the user notices any discrepancies in the transcribed text, they can rerecord their food intake. On the text arm, once user text is detected, base2Diet immediately queries Nutritionix to retrieve the stated foods’ nutritional content before creating a time-stamped diet log in Evernote.

### Personalized Alerts

To enhance user experience and increase engagement, we personalized all alerts. We used our custom server and cloud database with user information to personalize and send alerts. Each text notification on the base2Diet app addresses the user by name and refers to the appropriate time of day and the relevant meal for that time of day. For example, a breakfast alert for a participant named Jessica would present a text notification that says, “Good morning Jessica! What’s for breakfast?” To protect the user’s privacy, especially in public spaces, voice alerts did not mention the user’s name. Instead, they used prerecorded artificial intelligence–generated prompts [28] residing on the server. The user’s name would only be mentioned in the interactive dialogue between the user and the base2Diet app if the user is attempting to log their food intake using voice and the base2Diet app does not detect any speech. If no diet log is created 30 minutes after an alert is sent, only 1 additional reminder is sent to prevent intrusive app behavior. If food intake is logged before the set time for the reminder, base2Diet does not send any alerts. To support user privacy and freedom (the ability of a user to choose to exit unwanted actions or opt out of using any feature that is not of interest to the user at any given time, for example, voice alerts when a user is busy), base2Diet allows users to deactivate voice alerts for up to 1 full day. The system resets and reenables voice alerts every day at 4 AM. After each reset, users can manually deactivate the alerts for that day within the base2Diet app. When a user deactivates voice alerts, that user will receive text-only notifications but can use voice for food intake logging if they choose so.

### Autopopulation of Nutritional Content

The base2Diet app leverages Nutritionix’s NLP API to convert the natural language description of the consumed food provided by the user in text or speech form into its nutritional information through food item retrieval from the Nutritionix database. Consequently, the base2Diet app logs the food and nutritional content into an Evernote-based food diary.

### Data Collection Procedure

Flyers, social media, and word of mouth were used to recruit participants, who were assigned to either the text study arm (n=9) or the voice study arm (n=9). Though participants were located across the United States and were free to travel worldwide, the base2Diet app could detect their time zones and send alerts at the correct times. In the first phase of the study (phase I), all study participants received alerts at 9 AM, 12 PM, and 6 PM for the first 14 days. In the subsequent 14 days (phase II), participants were free to choose the times to receive the diet logging prompts, which they could change at any time during these 14 days.

On multiple occasions, we observed up to 5 distinct diet logs created by a user within a minute. Therefore, we defined distinct diet logging events as diet logging times separated by at least 30 minutes and otherwise considered multiple logs within a 30-minute span to be a part of a single-diet logging event. We defined an active day as a day when a participant creates at least 1 diet log by using the base2Diet app. Daily active users were defined as the number of users who log their food intake at least once per given study day. In phase II of the study, participants could choose when they wanted the app to remind them to log their food intake. We tracked how often users on both arms changed their alert times using Cloud Firestore [29]. We also enabled the participants to modify their diet logs at any time during the study and tracked this variable as well. Attrition rate was the rate at which participants dropped out of the study, defined as at least 7 consecutive days without the use of the base2Diet app. At the end of the study, all 18 participants completed a web-based usability survey that we developed, which had 14 multiple-choice questions, and 6 and 9 free-response questions in the text and voice arms, respectively. The survey was aimed at gauging participants’ perceptions of the usability and acceptability of voice technologies and NLP for automated diet capturing.

### Statistical Analysis

The response variables included (1) the total number of distinct diet logging events per participant, and (2) the total number of active days per participant. The normality of these variables was assessed graphically as well as formally by using the Shapiro-Wilk normality test. A comparison between means of the voice and text arm was performed through unpaired *t* tests, with a significance threshold of *P*<.05. All statistical analyses were performed in R (version 4.0.2), and all graphs were generated in R (version 4.0.2) using ggplot2.

### Ethics Approval

This study obtained ethical approval from the institutional review board at Duke University (protocol 2022-0236). Prior to enrollment, we obtained electronic informed consent from each study participant using REDCap.

All participants were provided a US $10 iTunes gift card as compensation for participating in the study. The disbursement of the gift cards was done at the end of the study after participants had filled out the exit survey.

## Results

### Participant Characteristics

There were 18 participants in the study (8 males, 10 females; age range 20-39 years). A total of 16 participants were enrolled at Duke University as undergraduate or graduate students, 1 was a University of North Carolina student, and 1 was a working professional. Six of the participants were identified as Black, 8 identified as Asian, and 5 identified as White. The study inclusion criteria were being older than 18 years, currently living in the United States, being proficient in written and spoken English, owning an iPhone, and not maintaining a food diary at the start of the study.

### User Engagement Metrics

**Figure 4:**
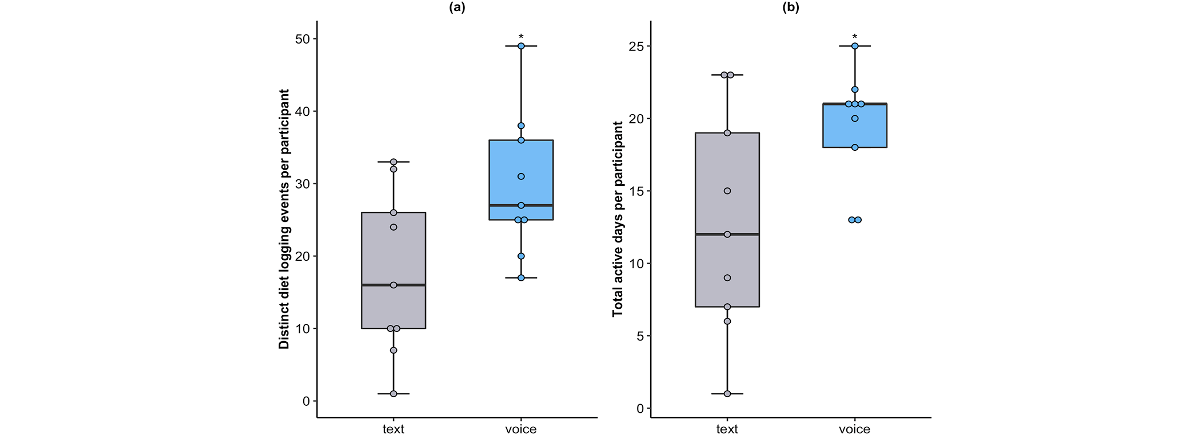
Box plot (A) shows the number of distinct diet logging events per participant by study arm (*P*=.03, unpaired *t* test). (B) shows the total active days per participant by study arm (*P*=.04, unpaired *t* test).

**Figure 5:**
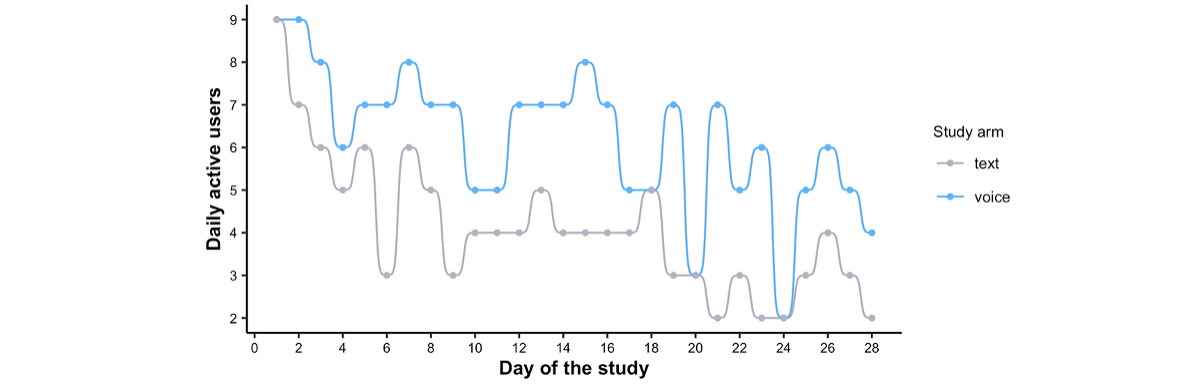
Total number of participants using the base2Diet app on each day of the study.

To understand the differences between the impact of text versus voice alerts on diet logging behaviors, we used seven key performance indicators and user engagement metrics: (1) distinct diet logging events per participant, (2) total active days per participant, (3) daily active users, (4) feature usage, (5) attrition rate, (6) diet log modification, and (7) perceived usability. The number of distinct diet logging events per participant was higher in the voice arm than in the text arm (mean 30 vs 18, respectively; *P*=.03, unpaired *t* test; Figure 4). In addition, the total number of active days per participant was also higher in the voice arm than in the text arm (mean 19 vs 13, respectively; *P*=.04, unpaired *t* test; Figure 4). In general, the number of daily active users was higher in the voice arm as compared with the text arm (mean 6 vs 4), with the exception of 3 of the study days in which the number of active users in the voice arm was equal to that of the text arm (Figure 5).

The attrition rate in the text arm was higher than in the voice arm, with only 1 (11%) participant dropping out of the study in the voice arm and 5 (56%) in the text arm (Figures S1 and S2 in Multimedia Appendix 1).

We also explored the influence of preselected versus user-selected alert timing for diet logging reminders. Although the first 2 weeks of the study used preselected alert times based on typical US meal times of 9 AM, 12 PM, and 6 PM [30], in the second 2 weeks of the 4-week study, users selected their alert times, and had the option to change the alert times at any point over those 2 weeks. Of the 18 participants, 4 participants in the voice arm changed their alert times beyond the initial setup, whereas none of the text arm participants changed their alert times after the initial setup (Table 1).

All 18 study participants completed the usability survey that was disseminated at the end of the study. Our survey analysis showed that 67% (n=12) of the participants highly valued the app’s functionality of automatically populating the food items’ nutritional content while also allowing the participants to edit the created diet logs. Interestingly, however, no participant actually modified their diet logs during the study, even though all participants viewed the logs and had the option to change them. Additionally, 83% (n=15) of the participants either agreed or strongly agreed that hands-free speech-based diet logging makes it easier to adopt diet-monitoring habits (Figure S3 in Multimedia Appendix 1). When text arm participants were asked if they would have appreciated personalized voice alerts to remind them to log their meals, 56% (n=5) of them said yes (Figure S4 in Multimedia Appendix 1). When asked to elaborate, 1 participant said, “I find it harder to be on top of logging my diet with only text alerts.” When voice arm participants were asked about their perception of voice alerts, 78% (n=7) of them either agreed or strongly agreed that they liked voice alerts, but they also valued the ability to turn them off when they were busy (Figure S4 in Multimedia Appendix 1). When asked to elaborate, 1 participant said, “Voice alerts made me have regular meals, which was good. I now kinda look forward to the prompts so that I know it’s time for a meal.”

**Table 1:** Number of participants who changed their alert times per study arm.

	Number of participants who changed alert times N times			
	0 times	1 time	5 times	15 times
Voice Arm	5	2	1	1
Text Arm	9	0	0	0

## Discussion

### Principal Findings

The primary objective of this study was to investigate the usability and acceptability of speech recognition technologies in automated diet logging. We designed and developed base2Diet—an iOS smartphone app for voice-assisted and automated diet logging. The results from the 28-day pilot study of base2Diet demonstrate that speech recognition technologies have tremendous potential to improve user experience and adherence to diet logging. Strikingly, the global market value for conversational artificial intelligence—chatbots or digital assistants that users can talk to—is expected to grow by 270%, from ≈US $6.8 million to ≈US $18.4 million between 2021 and 2026 [31]. The power of voice technologies to enhance user experiences and increase user engagement is among the key drivers of this growth. Our study, though a pilot in nature, supports the prevailing narrative, and our findings demonstrate that voice arm participants may be more likely to engage fully with the app than text arm participants (Table 1). Furthermore, a growing body of research points to the potential of voice technologies in improving diet recall and user experiences in diet monitoring [32-35]. For example, a study by Liang et al [35] revealed that 65% of younger and 60% of older participants preferred voice-assisted over web-based food recall. Beyond diet monitoring, voice technologies are being explored in numerous other domains [36-38], including assisted living [39].

Though the results of this study point to the promise of speech recognition technologies in automated diet logging, especially for improving user engagement and adherence, there is a need for user-centered design [40] to capture critical user needs, especially those pertaining to privacy and freedom. That 78% (n=7) of the participants in the voice arm either agreed or strongly agreed that they liked voice alerts, but also appreciated the ability to turn voice alerts off when busy (Figure S4 in Multimedia Appendix 1), reinforces previous findings that technologies should be adaptable to users’ preferences and the needs of the users should be central to the design process for technologies to be widely adopted. One participant’s experience clarifies this point. While elaborating why they appreciated speaking into their device to log their food intake, the participant said,

Dictating my meals to my phone was very convenient and took significantly less effort than typing. I love it as an interface, but I also loved having the option to type for [sic] when I was near others.

Follow-up conversations with participants revealed that all 18 participants viewed their diet logs at some time during the study. Although all participants had the option to manually edit their diet logs, none did it. We cannot be sure of why no one edited their diet logs; however, we do know that the average user inherently trusts autopopulated data or may not think it is worth the effort to change it, particularly when it comes to mobile apps [41,42]. Notably, the Instant Blood Pressure smartphone app [41], which measures blood pressure inaccurately, remained popular despite a disclaimer warning against its use for medical purposes. This highlights the significant influence that technology creators have over their users. As technology continues to become more ingrained in our daily lives, it is imperative for technology creators to acknowledge their ethical responsibility. Since the average user tends to trust information received from a mobile app without question, technology creators must ensure that the information and experiences they provide are accurate, nonharmful, and nonmisleading. Essentially, they must act ethically and prioritize the well-being of their users. Hence, it is crucial to raise awareness of sources of errors in autopopulated data to avoid errors being overlooked, particularly concerning app data used for health purposes.

### Limitations

Apple’s speech recognition framework [26], which we used in the base2Diet app, is imperfect. Several participants, especially those with foreign accents, pointed out that the speech recognition framework was not always able to accurately transcribe their voices. This issue has also been brought to light by many other researchers [43,44], and there is a recognized need to improve the existing speech recognition frameworks to improve their translational impact. Continuously deploying applications that use these frameworks that perform poorly among minoritized populations may result in those groups of people rejecting such technologies due to an inherent assumption that they will not work for them. This has the potential to further exacerbate existing inequities in health and wellness.

Though Nutritionix has a comprehensive database containing 821,036 grocery items, 186,168 restaurant items, and 10,351 common food items, not every possible food item is represented [45]. Several researchers have highlighted the need for more comprehensive food databases [19,46] to improve outcomes and enhance user experiences within automated diet logging applications. This gap also points to the potential of machine learning methods capable of imputing nutritional content from the previously unseen food items [47].

In addition to the highlighted technology issues, this work has several other limitations. First, our study population was small and limited to relatively young adults. Other populations, such as working individuals, elder individuals, and individuals with type 2 diabetes, should be involved in future studies exploring voice-assisted diet logging technologies. In addition, individuals needed to own an iPhone to participate in the study, which might have inhibited participation by those from lower-income brackets since iPhones tend to be more expensive than mobile phones running other operating systems such as Android. Finally, while hands-free technologies facilitate convenient diet logging, primarily when users have limited or no hand function or are engaged in other activities, the base2Diet app could not provide a completely hands-free experience because Apple’s iOS operating system restricts the ability to unlock the phone using voice.

### Future Research

Building on the promising results of this pilot study, there are several avenues for future research. One possible direction is to conduct large-scale, long-term trials with diverse populations, including individuals with specific dietary restrictions, or health conditions. This would provide a broader understanding of the generalizability and effectiveness of voice-assisted automated diet logging technologies in real-world settings.

Additionally, further investigations could be conducted to optimize the accuracy and efficiency of speech recognition technologies in capturing diet information, and to explore the integration of NLP techniques in food databases for a better understanding of user input. Further exploration of user preferences, needs, and privacy concerns in designing and developing these technologies could also provide valuable insights for optimizing user experience and adherence.

Furthermore, exploring the potential of voice alerts and just-in-time reminders as behavior change interventions could be an exciting area for future investigation. Overall, continued research in these areas has the potential to revolutionize the field of nutritional monitoring and interventions, improve public health outcomes, and address diet-related diseases such as type 2 diabetes.

### Conclusions

This study paves the way for further research on integrating speech recognition and NLP in automated diet logging. We developed a proof-of-concept mobile app that demonstrates the possibilities of hands-free, in-the-moment, voice-assisted diet logging. Additionally, we explored interactive voice alerts and identified the need for respecting user privacy and freedom, further validating existing knowledge around the need to understand user preferences or needs and center the design process around the user.

Our findings indicate that voice-assisted automated diet logging technologies may lead to more effective nutritional monitoring and interventions for numerous diet-related diseases such as type 2 diabetes. Existing research has already established that diet monitoring can reverse the progression of type 2 diabetes or prevent or delay its onset in individuals with prediabetes [48-50]. Voice assistants and just-in-time reminders are additional technological layers that can improve the accuracy and effectiveness of diet-monitoring technologies and ultimately promote better health outcomes.

## Appendix

Multimedia Appendix 1 Supplementary materials.

## Abbreviations

API: application programming interface
NLP: natural language processing

## Data Availability

Deidentified data sets generated during this study, and deidentified survey responses are available from the corresponding author upon request. All correspondence and requests for study material should be directed to JD.
