# Supplementary material for: Automated Diet Capture Using Voice Alerts and Speech Recognition on Smartphones: Pilot Usability and Acceptability Study
Source: JMIR Form Res. 2023 May 16;7:e46659. doi: 10.2196/46659 (PMC10230351; doi:10.2196/46659)
Supplement: Multimedia Appendix 1 [file formative_v7i1e46659_app1.pdf]

Appendix

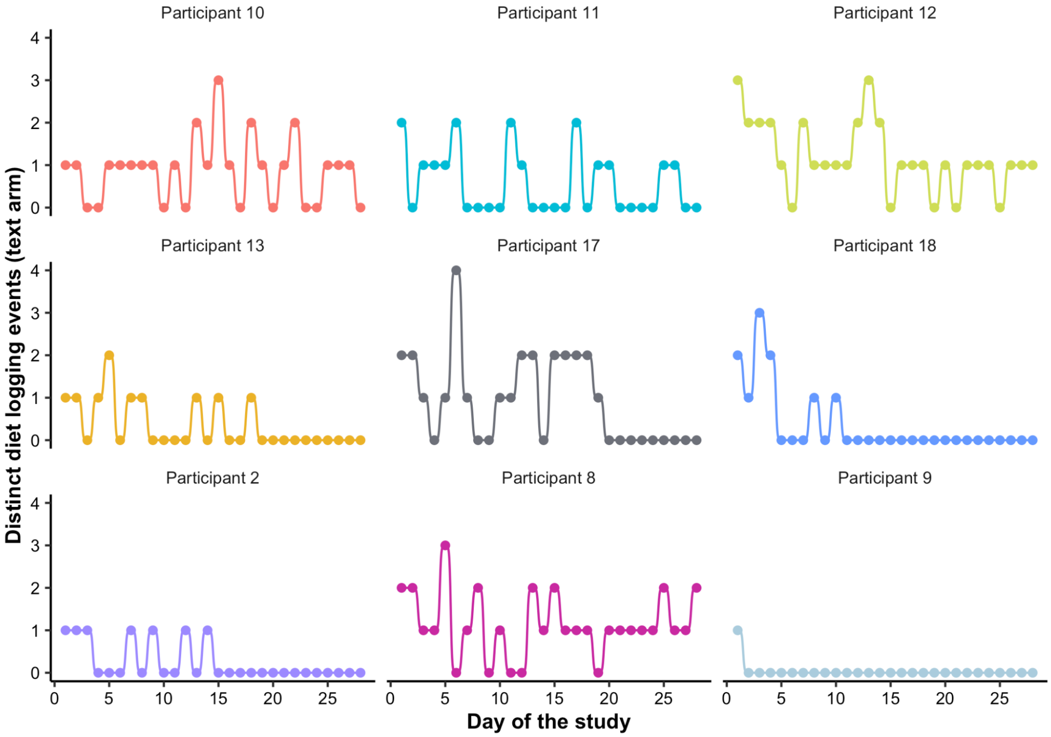

Figure S1. Distinct diet logging events per day for each participant in the text arm

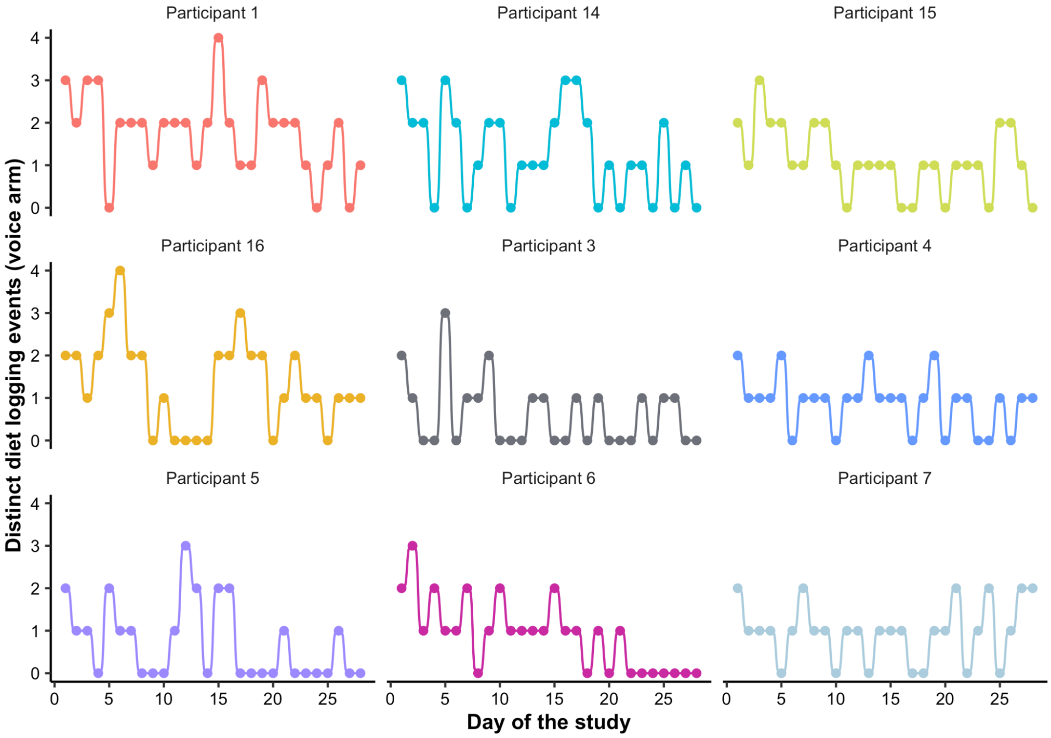

Figure S2. Distinct diet logging events per day for each participant in the voice arm

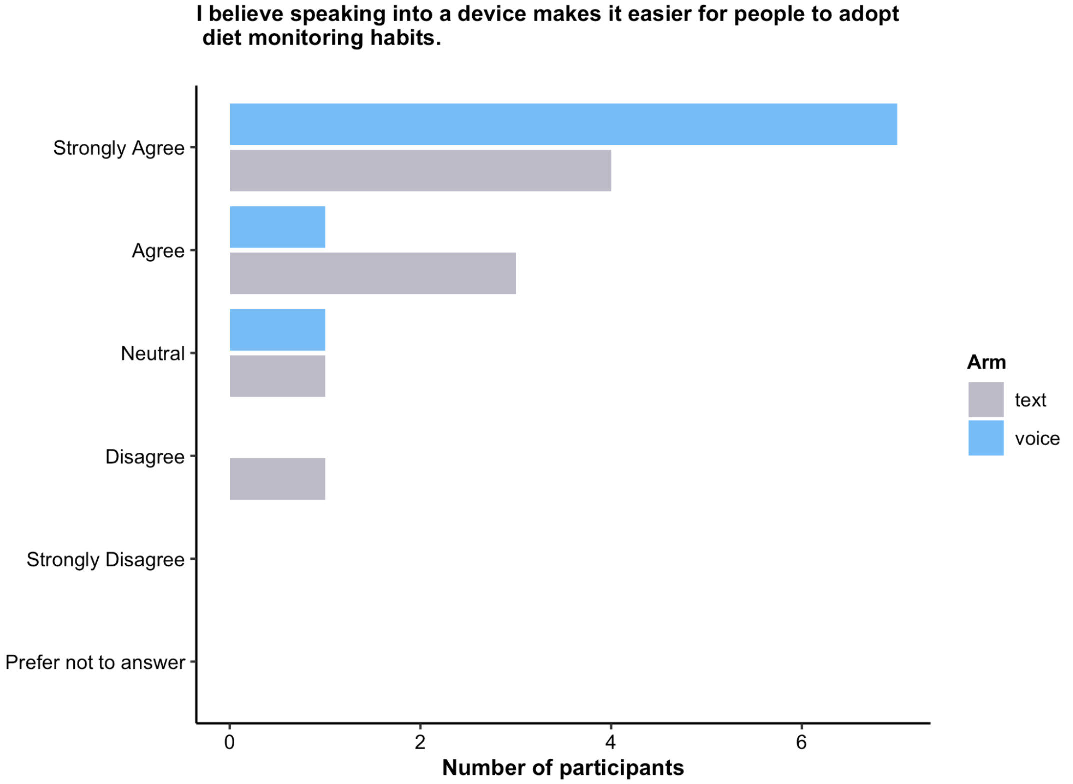

Figure S3. Exit survey response highlighting how participants in both arms believe speaking into a device (voice-assisted diet logging) is easier for people to adopt diet monitoring habits

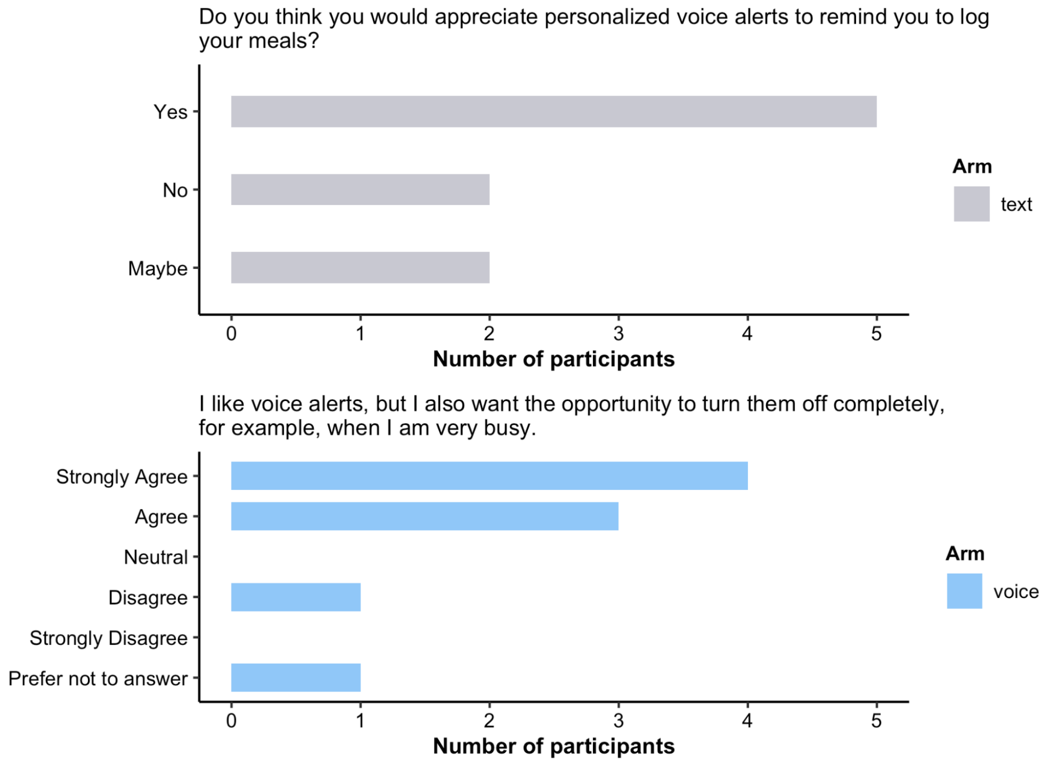

Figure S4. Exit survey response highlighting participants' perceptions towards voice alerts

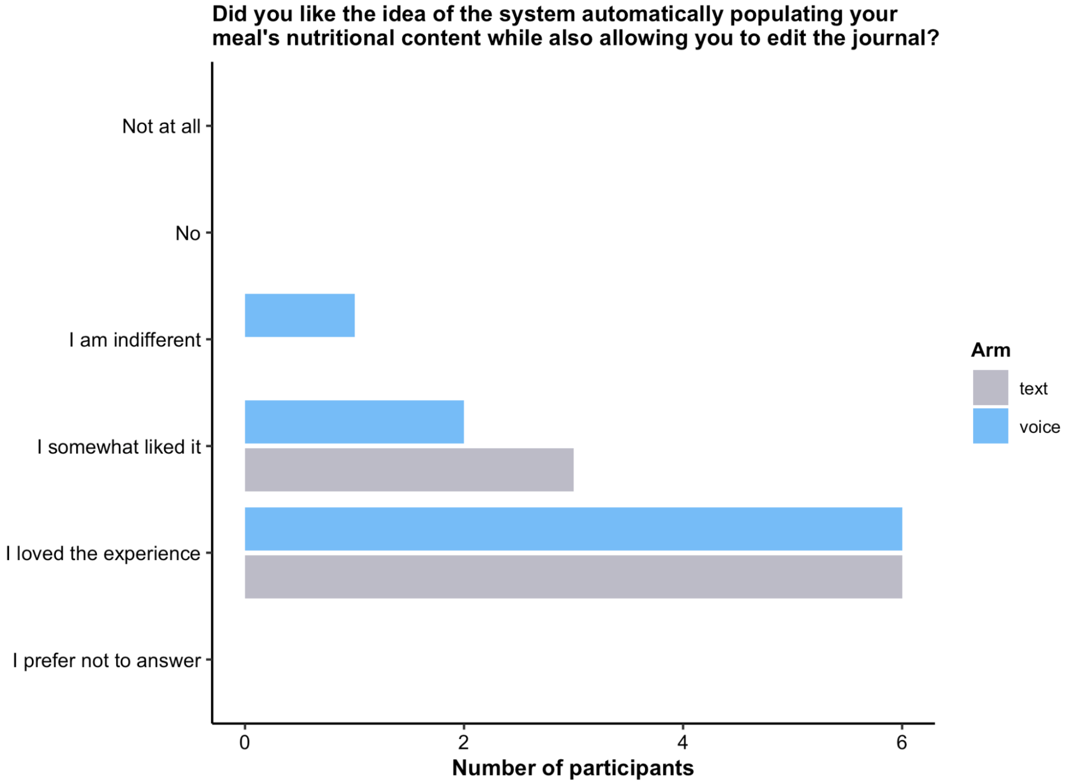

Figure S6. Exit survey response highlighting how participants in both arms like the idea of the automatic population of a meal's nutritional content while also allowing them to edit the journal

Text Arm Exit Survey Questions

1) Please give your overall rating of the quality of this study (e.g., approach, experience, research impact)
    a. Very poor
    b. Poor
    c. Adequate
    d. Good
    e. Excellent
    f. I prefer not to answer

2) Please elaborate on your experience.

3) Do you think you would benefit from a voice-based system that provides voice alerts, and allows you to speak into your device to log your diet?
    a. Yes
    b. No
    c. Maybe

4) Which of the two platforms do you think you will prefer?
    a. Voice-based platform
    b. Text-based platform
    c. I prefer not to answer

5) Please elaborate.

6) Do you think you would appreciate personalized voice alerts to remind you to log your meals?

a. Yes
b. No
c. Maybe

7) Do you think you would like to speak into your phone to capture your diet?
    a. Not at all
    b. No
    c. I am indifferent
    d. I somewhat liked them
    e. I loved them
    f. I prefer not to answer

8) Please elaborate on your experience.

9) Would you recommend voice alerts for future diet capturing frameworks?

10) Please elaborate.

11) Did you like the idea of the system automatically populating your meal's nutritional content while also allowing you to edit the journal?
    a. Not at all
    b. No
    c. I am indifferent
    d. I somewhat liked that
    e. I loved the experience
    f. I prefer not to answer

12) If the system was automatically populating your meal's nutritional content without allowing you to edit, would that be okay with you?
    a. Not at all
    b. No
    c. I am indifferent
    d. Somewhat
    e. Yes
    f. I prefer not to answer

13) What are some improvements you envision for the system? What would make you adopt dietary monitoring habits?

The following table is used for answering questions 14-19.

Strongly Agree	Agree	Neutral	Disagree	Strongly Disagree	N/A

14) I believe this kind of research is useful.
15) I would like to see more applications with voice alerts.
16) I like voice alerts, but I also want the opportunity to turn them off completely, for example, when I am very busy.
17) I believe speaking into a device makes it easier for people to adopt diet monitoring habits.
18) I believe food logging is important.
19) I enjoyed this study.

20) Is there anything that would have been helpful for you as you participated in the study?

Voice Arm Exit Survey Questions

1) Please give your overall rating of the quality of this study (e.g., approach, experience, research impact)
   a. Very poor
   b. Poor
   c. Adequate
   d. Good
   e. Excellent
   f. I prefer not to answer

2) Please elaborate on your experience.

3) Which of the two platforms did you like best?
   a. Voice-based platform
   b. Text-based platform
   c. I prefer not to answer

4) Please elaborate on your experience.

5) Did you like the voice alerts?
   a. Not at all
   b. No
   c. I am indifferent
   d. I somewhat liked them
   e. I loved them
   f. I prefer not to answer

6) Please elaborate on your experience.

7) Would you recommend voice alerts for future diet capturing frameworks?

8) Please elaborate if there are any refinements you would make or you would leave things as is.

9) Did you like speaking into your phone to capture your diet?
   a. Not at all
   b. No
   c. I am indifferent
   d. I somewhat liked that
   e. I loved the experience
   f. I prefer not to answer

10) Please elaborate on your experience.

11) Would you recommend speaking into your phone for future diet capturing frameworks?

12) Please elaborate if there are any refinements you would make, or if you would leave things as is.

13) What would you change about any of the two systems?

14) Did you like the idea of the system automatically populating your meal's nutritional content while also allowing you to edit the journal?

a. Not at all
b. No
c. I am indifferent
d. I somewhat liked that
e. I loved the experience
f. I prefer not to answer

15) If the system was automatically populating your meal's nutritional content without allowing you to edit, would that be okay with you?

a. Not at all
b. No
c. I am indifferent
d. Somewhat
e. Yes
f. I prefer not to answer

16) What are some improvements you envision for the system? What would make you adopt dietary monitoring habits?

The following table is used for answering questions 17-22.

Strongly Agree	Agree	Neutral	Disagree	Strongly Disagree	N/A

17) I believe this kind of research is useful.
18) I would like to see more applications with voice alerts.
19) I like voice alerts, but I also want the opportunity to turn them off completely, for example, when I am very busy.
20) I believe speaking into a device makes it easier for people to adopt diet monitoring habits.
21) I believe food logging is important.
22) I enjoyed this study.

23) Is there anything that would have been helpful for you as you participated in the study?
